# Drug Resistance of *Mycobacterium tuberculosis* Based on Whole-Genome Sequencing in the Yi Ethnic Group, Sichuan Province, China

**DOI:** 10.1155/2023/4431209

**Published:** 2023-01-23

**Authors:** Wenfeng Gao, Xiaoru Chen, Liang Yao, Jing Li, Yuan Gao, Ting Li, Yunkui Li, Weina Wang, Shu Zhang, Jinge He

**Affiliations:** ^1^Institute for Tuberculosis Control and Prevention, Sichuan Center for Disease Control and Prevention, Chengdu, Sichuan, China 610041; ^2^Laboratory Medicine, Liangshan Yi Autonomous Prefecture Center for Disease Control and Prevention, Liangshan, Sichuan, China 615000; ^3^Laboratory Medicine, Butuo County People's Hospital, Liangshan, Sichuan, China 616350

## Abstract

This study investigated drug-resistant tuberculosis (DR-TB) in the Yi ethnic group. The study was designed to identify risk factors for DR-TB and its relationship with HIV/AIDS. To establish the resistance to antituberculosis drugs, whole-genome sequencing (WGS) was performed using culture-positive *Mycobacterium tuberculosis* samples collected from people of the Yi ethnic group from March 2019 to March 2021. Baseline characteristics were obtained from China's tuberculosis surveillance system. A total of 116 *M. tuberculosis* strains were included in the final analysis. Lineage 2.2 (75.86%) was the dominant sublineage, followed by lineage 4.5 (18.97%) and lineage 4.4 (5.17%). The rates of rifampicin-resistant (RR-TB), multidrug-resistant (MDR-TB), and preextensively drug-resistant TB (pre-XDR-TB) were 18.97%, 10.34%, and 6.03%, respectively. Drug-resistant strains were not found in the elderly (age ≥ 65 years). The proportions of RR/MDR-TB and pre-XDR-TB cases among re-treatment patients were higher than those among new patients (*χ*^2^ = 12.155, *P* = 0.003; *χ*^2^ = 22.495, *P* = 0.001, respectively). The pre-XDR-TB case proportions were higher among female patients than among males and higher among referred patients (*χ*^2^ = 5.456, *P* = 0.032; *χ*^2^ = 15.134, *P* = 0.002, respectively). The rates of RR/MDR-TB and pre-XDR-TB did not differ appreciably among groups with different HIV infection statuses nor lineage populations. DR-TB poses a serious challenge to the Yi ethnic group. Re-treatment patients, women, and referred patients were at high risk of MDR/RR-TB or pre-XDR-TB while HIV and lineage 2 had negligible association with drug resistance. Whole-genome sequencing should be used to guide the design of treatment regimens and to tailor public interventions.

## 1. Introduction

Tuberculosis (TB) is a chronic communicable disease and remains a serious public threat worldwide. Although the global burden is stable, drug-resistant TB (DR-TB) is a major threat to the WHO End TB Strategy due to the limited treatment options, longer treatment duration, lower cure rate, and the need for more second-line anti-TB drugs with greater side effects and higher prices [1]. According to the WHO, China is estimated to have the second-highest incidence of TB; multidrug-resistant TB or rifampicin-resistant TB (RR/MDR-TB) is only lower than India [2].

Liangshan Yi Autonomous Prefecture (Liangshan) of Sichuan province, located in southwest China, is a mountainous area populated by the Yi ethnic group and has an underdeveloped economy. Our previous study found that the TB epidemic is particularly serious in the Yi ethnic group, especially in regions with high HIV/AIDS prevalence [3, 4]. Butuo County (Butuo) is one of the counties in Liangshan most plagued by HIV/AIDS; it reportedly has the highest incidence of the disease in China, according to a county-level based survey. However, no study has investigated the situation of DR-TB and its relationship with HIV/AIDS in the Yi ethnic group.

The culture-based phenotypic drug sensitivity test, which detects the growth of *Mycobacterium tuberculosis* in the presence of anti-TB drugs, is limited by the slow mycobacterial duplication time and lack of a uniform minimum inhibitory concentration analysis or evaluation. Genotypic laboratory tests can quickly detect the presence of DNA mutations conferring resistance but can only identify a limited number of resistance genes. Recently, whole-genome sequencing (WGS) has been widely used in testing for drug resistance because it offers more accurate prediction and better determination of resistance to most drugs. WGS also has high predictive power to infer resistance profiles in areas with high TB burdens [5]. Furthermore, WGS can be used to understand evolution, lineages, and genomic variations [6]. Compared with analysis of the restriction fragment length polymorphisms of the IS*6110* gene (IS*6110*-RFLP), spacer oligonucleotide typing (spoligotyping), and mycobacterial interspersed repetitive unit-variable number of DNA tandem repeats, WGS provides high-resolution data that can be used to determine genetic relationships, especially between strains that are very close at the genetic level [7, 8].

In this study, we chose Butuo County as the research site to investigate the genetic diversity and drug-resistant profile of local circulating strains in the local Yi ethnic group, using WGS for the first time, to provide a scientific basis for TB prevention and control.

## 2. Materials and Methods

### 2.1. Sample Collection

This study was conducted from March 2019 to March 2021 at Butuo County People's Hospital. Patients with pulmonary tuberculosis (PTB) were diagnosed following the “Criteria for PTB Diagnosis” of China (WS288-2017). All cases of PTB among the Yi ethnic group were included in this research. Patients from other ethnic groups were excluded. Sputum samples were collected from all study participants. Each sample was liquefied using 4% NaOH and inoculated into tubes with acidified Löwenstein-Jensen medium for further culture. The identification of *M. tuberculosis* included *p*-nitrobenzoic acid testing. Subsequently, *M. tuberculosis* strains were tested for WGS.

### 2.2. Patient Information

Demographic information (gender, age, occupation, and census register) and clinical characteristics of patients (HIV infection status, history of previous TB treatment, visiting hospital delay, health system delay, and patient source) were obtained from the Tuberculosis Information Management System of China. Visiting hospital delay was defined as the time interval between the date of symptom onset and the patient's first consultation with a TB-designated hospital, and health system delay was defined as the time interval between the patient's first consultation with TB-designated hospitals and the date of diagnosis. Pulmonary TB patients were detected via clinic visits due to symptoms, referral, tracing, or health examination.

### 2.3. Whole-Genome Sequencing

Extraction and purification of genomic DNA were carried out following Bacterial DNA Extraction Kit (Gene-Optimal, 60300 K-50 T) protocols. Libraries were constructed on the Illumina platform using an FS DNA Lib Prep Kit V6 (RK20259). The samples were sequenced using an Illumina NovaSeq 6000 sequencer. Base-calling was performed using PE 150 software. All whole-genome sequencing procedures were performed by Shanghai Gene-Optimal Science & Technology Co. Ltd. (Shanghai, China). Cutadapt (v1.15) was used to trim adapter sequences at the tail of sequencing reads. Then, sequencing reads were aligned to reference genome (H37Rv, NC000962.3) using BWA (v0.7.15). Duplicated reads were marked by Picard (v2.4.1). Qualimap (v2.2.1) was used to calculate base quality metrics, genome mapping rate, and the coverage of targeted regions. *Mycobacterium tuberculosis* samples were selected for variant detection and annotation. The selection criteria were as follows: detected by 100% coverage of reads mapping to *M. tuberculosis* specific sequences (H37Rv region 315947-316534), with an average depth > 5. SNP and InDel calling was performed following the SAMtools (v1.6) and VarScan (v2.3.9). Large deletions were detected using Delly (v0.8.7). Variant filtering and annotating were done using a finely tuned in-house script. The WGS data obtained in our study were compared with available databases of *M. tuberculosis* drug resistance-associated gene mutations [9, 10]. Strains with a sequence depth less than 20× or a genome coverage less than 95% were excluded from the analysis. The phylogenetic tree was constructed using the maximum likelihood method and reconstructed using RAxML-NG12 (v.1.0.2) with “–model GTR + G + ASC_LEWIS”. All strains were phylogenetically classified according to the SNPs barcode nomenclature proposed by Napier G [11]. The phylogenetic tree was visualized and modified using iTOL (https://itol.embl.de/).

### 2.4. Statistical Analysis

Chi-squared or Fisher's exact tests were used for categorical data. Correlation analysis was performed to explore the risk factors for high-drug resistance. All statistical analyses were performed in SPSS version 20 software (SPSS Inc., Chicago, Illinois.). *P* values less than 0.05 were considered statistically significant.

### 2.5. Definitions

Rifampicin-resistant (RR-TB) or RR/MDR-TB was classified as *M. tuberculosis* resistance to at least rifampicin. MDR-TB was classified as *M. tuberculosis* resistance to at least isoniazid and rifampicin. Pre-XDR-TB was defined as MDR-TB with additional resistance to any fluoroquinolones (moxifloxacin or ofloxacin), based on a new definition released by WHO in January 2021 [12].

### 2.6. Ethics Approval

The study protocol was approved by the Ethical Review Committee at the Biomedical Ethics Committee, West China Hospital, Sichuan University (No. 2019-151). All participants provided written informed consent after reviewing the description of the study.

## 3. Results

### 3.1. Demographic and Clinical Characteristics

A total of 146 positive cultures were collected from among 784 Yi ethnic group patients reported from March 2019 to March 2021 at Butuo. Of these, 133 cultures were detected by WGS, while 13 isolates were not *M. tuberculosis*. Seventeen isolates were excluded due to the failure of WGS. Therefore, 116 *M. tuberculosis* strains were included into the final analysis. Seventy-nine patients (68.10%, 79/116) were males. The median age of the PTB patients was 38 (range 7–83) years. The majority of PTB cases (91.38%, 106/116) were newly diagnosed, while 10 (8. 62%) cases had received previous treatment. The percentage of patients who were HIV-infected was 34.48% (40/116). Most patients were native residents (94.39%, 101/116). The median health system delay was 2.5 days. The median visiting hospital delay was 35 days. The vast majority of patients were found by tracing. Detailed demographic information and clinical characteristics of the study population are shown in Table 1.

### 3.2. Phylogenetic Analysis

**Figure 1:**
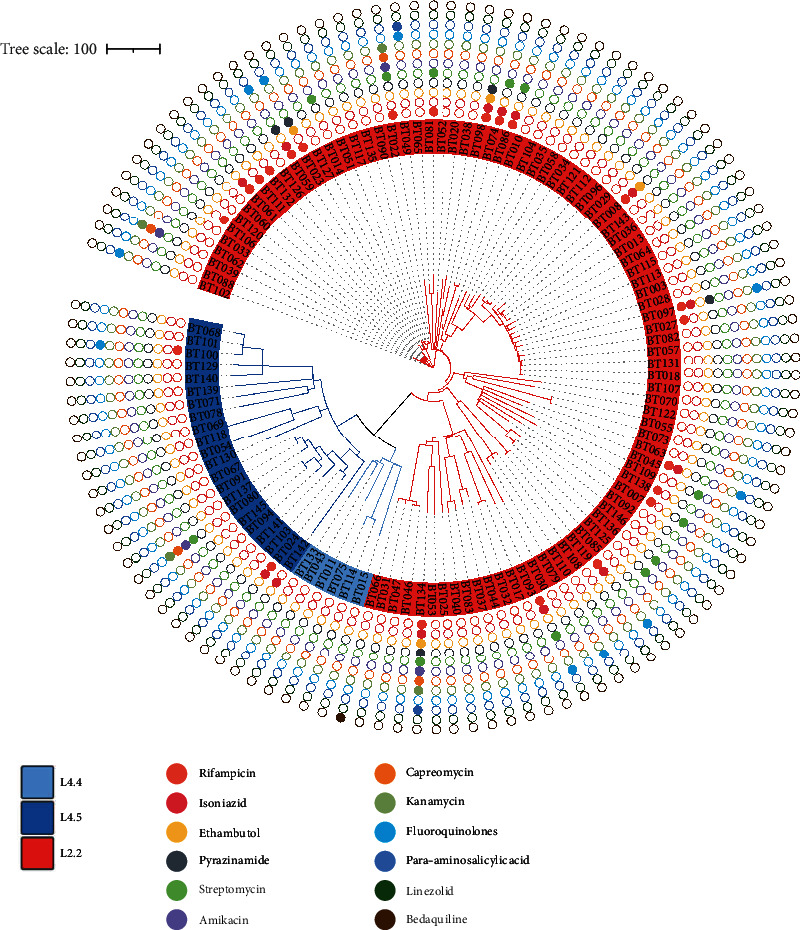
Phylogenetic tree for 116 *M. tuberculosis* isolates from the Yi ethnic group. From inside to outside, the solid-coloured circles represent drug resistance mutations for rifampicin, isoniazid, ethambutol, pyrazinamide, streptomycin, amikacin, capreomycin, kanamycin, fluoroquinolones, para-aminosalicylic acid, linezolid, and bedaquiline.

The phylogenetic tree revealed that three lineages of *M. tuberculosis* isolate circulated from the Yi ethnic group (Figure 1). Two main lineages were identified: 75.86% (88/116) of strains were assigned to lineage 2 (East Asian genotype) and 24.14% (28/116) to lineage 4 (Euro-American genotype). Lineage 2.2 was the dominant sublineage, accounting for 75.86% (88/116) and was followed by lineage 4.5 (18.97%, 22/116) and lineage 4.4 (5.17%, 6/116).

### 3.3. Prediction of Drug Resistance and Mutation Characteristics

As predicted, one or more drug resistance mutations were detected in 33 strains, which means 28.45% of the strains were drug-resistant; the rest were considered as susceptible strains. In 116 strains, rifampicin-resistant (RR-TB), multidrug-resistant (MDR-TB), and preextensively drug-resistant (pre-XDR-TB) strains were found in 22, 12, and 7 patients, accounting for 18.97%, 10.34%, and 6.03%, respectively.

The RR-TB, MDR-TB, and pre-XDR-TB rates among new patients were 15.09% (16/106), 7.55% (8/106), and 2.83% (3/106), respectively. Meanwhile, those rates among re-treatment patients were 60.00% (6/10), 40.00% (4/10), and 40.00% (4/10), respectively. The RR-TB, MDR-TB, and pre-XDR-TB rates among HIV/AIDS patients were 25.00% (10/40), 15.00% (6/40), and 7.50% (3/40), respectively. In lineage 2 samples, the rates of RR-TB, MDR-TB, and pre-XDR-TB were 22.73% (20/88), 12.50% (11/88), and 6.82% (6/88), respectively. The drug-resistant strain was not found in elderly patients (≥65 years of age).

The resistances of 116 strains to different drugs are shown in Table 2. In this study, 12 strains resistant to antituberculosis drugs were detected, and the drug resistance rates for rifampicin (RIF: 22/116, 18.97%), isoniazid (INH: 13/116, 11.21%), streptomycin (Sm: 12/116, 10.34%), fluoroquinolones (10/116, 8.62%), and ethambutol (EMB: 8/116, 6.90%) were high. Mutations of 22 RIF-resistant strains were detected in the *rpoB* gene. The most common drug-resistant mutations were *rpoB*_S450L (4/22, 18.18%). Six (27.27%, 6/22) of the RIF-resistant strains had combined mutations at two loci. Three (13.64%, 3/22) strains harboured gene mutations in *RRDR*, namely, D545E, E156D, and K119N, respectively. Thirteen strains had INH gene mutations, with the majority of the mutations categorized as *katG*_S315N (30.77%, 4/13), *katG*_S315T (23.08%, 3/13), and *katG*_frameshift (15.38%, 2/13). Combined mutations at two loci were present in 15.38% (2/13) of the INH-resistant strains. The Sm resistance gene mutations occurred in *rpsL*, *rrs,* and *gid*, and a total of five resistance mutation types were generated, mainly *rpsL*_K88R (41.67%, 5/12). A total of ten mutation types were generated in the resistance genes of fluoroquinolones, mainly *gyr*_A90V (40.00, 4/10). Ethambutol resistance was found in eight strains and a total of five mutation types were produced; the main mutation types were *embB*_M306V (37.50%, 3/8). One (12.50%, 1/8) of the EMB-resistant strains has combined mutations at two loci. The resistance gene mutations to other drugs are shown in Table 2.

### 3.4. Characteristics of Drug Susceptibility

The proportions of RR/MDR-TB and pre-XDR-TB in re-treatment patients were higher than in new patients (*χ*^2^ = 12.155, *P* = 0.003; *χ*^2^ = 22.495, *P* = 0.001, respectively). The pre-XDR-TB proportion was higher among female patients (*χ*^2^ = 5.456, *P* = 0.032). The pre-XDR-TB rate was higher among referred patients than among patients from other sources (*χ*^2^ = 15.134, *P* = 0.002). RR/MDR-TB and pre-XDR-TB did not differ appreciably among groups with differing HIV infection status nor lineage populations (Table 3).

## 4. Discussion

To the best of our knowledge, this is the first study to use whole-genome sequencing technology to predict drug resistance and analyse genetic diversity of TB among members of the Yi ethnic group with high HIV/AIDS prevalence. Our findings help understand the drug resistance spectrum and toxicity of local epidemic strains and provide a scientific basis for TB prevention and control.

The epidemic situation of DR-TB in the Yi ethnic group is serious, with a 10.34% incidence rate of MDR-TB, higher than the average level in Sichuan province (8.14%) [13] and higher than that in other regions of China [14–17]. Furthermore, the MDR-TB rates among new and re-treated patients of the Yi ethnic group (7.55% and 40.00%, respectively) are much higher than the baselines reported in the national survey in 2007 (5.7% and 25.6%, respectively) [18] and higher than the global average in the last decade (3–4% and 18–21%, respectively) [2].

Although the Yi ethnic group already has a serious epidemic of drug-resistant TB, the situation has the potential to deteriorate further. The overall RR/MDR-TB rate was 18.97%, while the rates in new and re-treatment patients were 15.09% and 60.00%, respectively, which are higher than those reported in a previous Indian study [19]. In addition to MDR, 7.55% (8/106) of new patients and 20.00% (2/10) of re-treated patients were resistant to rifampicin. If these patients are not effectively treated, they can easily develop MDR-TB. In addition, 31.82% (7/22) of all RR-TB patients suffered from pre-XDR-TB, a proportion higher than the global rate (20%) [20] and higher than the Chinese rate (25%) [18]. These patients are easily susceptible to developing XDR-TB, which would further worsen the local TB control.

The most common drug-resistant mutations (RIF-, fluoroquinolone-, and EMB-resistant strains) in the Yi ethnic group were consistent with previous reports, but differed in the frequency of resistance. In terms of the MDR-TB strain in all China, the majority mutation types of INH and Sm resistance-associated genes were katG315T and rpsLK43R, respectively, which differed from the results of our study [21]. These differences may be related to the study sample size, strain genotype, medication habits, and patient compliance.

The results showed strong associations between pre-XDR-TB and treatment history, gender, and source of patients; meanwhile, MDR/RR-TB was associated with treatment history. In previous studies, treatment history has been reported to be the most influential risk factor for developing drug tolerance [22–24]. Patients with a history of antituberculosis treatments might have problems with irregular treatment or interruption of treatment, leading to increased selection pressure for drug resistance mutations and ultimately drug tolerance [24].

In addition, the proportion of pre-XDR-TB among female patients was higher than that in males, which was possibly related to lower incomes among females, resulting in poor treatment conditions, and the fact that females were more prone to irregular medication [25]. The pre-XDR-TB rate among referred patients was higher than that among patients from other sources. Patients referred for treatment in nondesignated medical institutions or outpatient clinics could not get the correct diagnosis nor standardized medication in a timely manner; in particular, abuse of fluoroquinolones may have occurred, resulting in the generation of pre-XDR-TB.

The global epidemiology of drug-resistant TB in HIV-infected persons is not known owing to a lack of information. The global project of antituberculosis drug resistance explored the interaction between HIV infection and drug-resistant TB in seven settings, none of which had a high prevalence of HIV infection. The results showed significant associations only in two settings, in Latvia and Ukraine [26]. Eldholm et al. and Masenga et al. reported that HIV coinfection was not significantly associated with drug-resistant TB [27, 28]. Our result for this study area with high prevalence of HIV infection echoed their finding.

In our study, the drug-resistant strain was not found in the elderly (aged at least 65 years), which was a similar result to that of the Indian study and may be explained by reduced survival among those with TB infections [19].

Twenty-three percent of the global TB burden is ascribed to lineage 2 strains, which are mainly distributed across East Asia and have been associated with drug resistance, hypervirulence, and high transmissibility [29]. The prevalent TB bacteria in the Yi ethnic group were lineage 2 (all Beijing genotype (lineage 2.2)), accounting for 75.21% of cases in this study, which is close to the average level of China (70%) [30]. However, lineage 2 was not the major contributor for drug resistance in our study, which was consistent with the conclusion of another study conducted in southern China [31]. More research is required to clarify the underlying reasons for the lack of association of lineage 2 with the prevalence of drug resistance TB in the Yi ethnic group.

## 5. Conclusions

In summary, the prevalent strains in the Yi ethnic group belonged to lineage 2, and our efforts to mitigate the impacts of TB should be focused on this genotype. The Yi ethnic group are facing a serious situation of DR-TB. Re-treatment patients were at high risk of MDR/RR-TB and pre-XDR-TB. Measures should be taken to reduce acquired drug resistance during treatment through standardizing therapy and management of patients. Women and referred patients were more susceptible to pre-XDR-TB. Those key populations should receive more attention in the management of TB. The drug-resistant strain was not found in the elderly (≥65 years of age). HIV coinfection and lineage 2 were not found to be significantly associated with drug-resistant TB. Whole-genome sequencing, as the most promising tool for predicting drug resistance and analysing genetic diversity, should be used to guide the design of treatment regimens and tailor public interventions.

## Figures and Tables

**Table 1:** Demographic information and clinical characteristics of 116 pulmonary tuberculosis cases in the Yi ethnic group, Sichuan, China.

Characteristic	Total cases (*N* = 116)		New cases (*N* = 106)		Re-treated cases (*N* = 10)	
	*N*	%	*N*	%	*N*	%
*Sex*						
Male	79	68.10	72	67.92	7	70.00
Female	37	31.90	34	32.08	3	30.00
*Age group*						
0–17	9	7.76	9	8.49	0	0.00
18–64	95	81.90	85	80.19	10	100.00
65–	12	10.34	12	11.32	0	0.00
*Occupation*						
Farmer	110	94.87	100	94.34	10	100.00
Other	6	5.17	6	5.66	0	0.00
*Census register*						
Resident	101	94.39	94	94.95	7	87.50
Migrant	6	5.61	5	5.05	1	12.50
*HIV infection status*						
Yes	40	34.48	35	33.02	5	50.00
No	76	65.52	71	66.98	5	50.00
*Health system delay*						
<14 days	87	81.31	80	80.81	7	87.50
≥14 days	20	18.69	19	19.19	1	12.50
*Visiting hospital delay*						
<14 days	35	32.71	33	33.33	2	25.00
≥14 days	72	67.29	66	66.67	6	75.00
*Patient source*						
Clinic visit due to symptoms	4	3.67	3	3.00	1	11.11
Tracing	92	84.40	86	86.00	6	66.67
Referral	2	1.83	1	1.00	1	11.11
Health inspection	11	10.09	10	10.00	1	11.11

Note: nine cases were missing information for census register, health system delay, and visiting hospital delay. Seven cases were missing information for patient source.

**Table 2:** Drug resistance and gene mutation type in 116 strains.

Drug	Resistant strains		Mutation		
	*N*	%	Type	*N*	%
RIF	22	18.97	*rpoB*_S450L	4	18.18
			*rpoB*_H445Y	3	13.64
			*rpoB*_D435Y	2	9.09
			*rpoB*_H445S	2	9.09
			*rpoB*_R225P; *rpoB*_S450L	2	9.09
			*rpoB*_D435V	1	4.55
			*rpoB*_D545E	1	4.55

INH	13	11.21	*rpoB*_E156D	1	4.55
			*rpoB*_H445V	1	4.55
			*rpoB*_K119N	1	4.55
			*rpoB*_K446Q; *rpoB*_D545E	1	4.55
			*rpoB*_P280L; *rpoB*_L452P	1	4.55
			*rpoB*_S450L; *rpoB*_D545E	1	4.55
			*rpoB*_P45A; *rpoB*_S450L	1	4.55
			*katG*_S315T	4	30.77
			*katG*_S315N	3	23.08
			*katG*_frameshift	2	15.38
			*inhA*_S94A	1	7.69
			*katG*_gene_deletion	1	7.69
			*katG*_S315N; *katG*_W90stop	1	7.69
			*fabG1*_T-8G; *ahpC*_C-57 T	1	7.69
Sm	12	10.34	*rpsL*_K88R	5	41.67
			*rpsL*_K43R	3	25.00
			*rrs*_C799T	2	16.67
			*rrs*_G888A	1	8.33
			*gid*_326_del_C	1	8.33
FQs	10	8.62	*gyrA*_A90V	4	40.00
			*gyrA*_D94G	2	20.00
			*gyrA*_D94N	1	10.00
			*gyrA*_D94A	1	10.00
			*gyrA*_A90V; *gyrA*_D94A	1	10.00
			*gyrB*_D461N; *gyrA*_A90V	1	10.00
EMB	8	6.90	*embB*_M306V	3	37.50
			*embB*_M306I	2	25.00
			*embB*_G406S	1	12.50
			*embA*_C-16G	1	12.50
			*embB*_M306I; *embB*_D354A	1	12.50
PZA	5	4.31	*pncA*_D63G	1	20.00
			*pncA*_D49A	1	20.00
			*pncA*_frameshift	1	20.00
			*pncA*_L151S; *pncA*_D63G	1	20.00
			*pncA*_G108R; *pncA*_D49N	1	20.00

Am	4	3.45	*rrs*_C1402A; *rrs*_G1484T	2	50.00
			*rrs*_C1402A	1	25.00
			*rrs*_A1401G	1	25.00
Cm	4	3.45	*rrs*_C1402A; *rrs*_G1484T	2	50.00
			*rrs*_C1402A	1	25.00
			*rrs*_A1401G	1	25.00
Km	4	3.45	*rrs*_C1402A; *rrs*_G1484T	2	50.00
			*rrs*_C1402A	1	25.00
			*rrs*_A1401G	1	25.00
PAS	2	1.72	*thyA*_H75N	1	50.00
			*folC*_E153A	1	50.00
BDQ	1	0.86	*Rv0678*_192_ins_G	1	100.00
LZD	0	0.00	/	/	/

Note: RFP: rifampicin; INH: isoniazid; EMB: ethambutol; PZA: pyrazinamide; Sm: streptomycin; Am: amikacin; Cm: capreomycin; Km: kanamycin; FQs: fluoroquinolones; PAS: para-aminosalicylic acid; BDQ: bedaquiline; LZD: linezolid.

**Table 3:** Demographic, bacteriological, and clinical characteristics of drug-resistant tuberculosis cases in the Yi ethnic group, Sichuan, China.

Characteristics	RR/MDR-TB (*N* = 22)				Pre-XDR-TB (*N* = 7)			
	*N*	%	*χ* ^2^	*P*	*N*	%	*χ* ^2^	*P*
*Sex*			2.297	0.137			5.359	**0.033**
Male	12	15.19			2	2.53		
Female	10	27.03			5	13.51		
*Age group*			58.632	0.188			57.802	0.209
0–17	2	22.22			1	11.11		
18–64	20	21.05			6	6.32		
65+	0	0.00			0	0.00		
*Occupation*			3.965	0.081			1.261	0.318
Farmer	19	17.27			6	5.45		
Other	3	50.00			1	16.67		
*Patient classification*			11.99	**0.003**			22.265	**0.001**
New cases	16	15.09			3	2.83		
Re-treated cases	6	60.00			4	40.00		
*HIV infection status*			1.447	0.319			0.231	0.691
Yes	10	25.00			3	7.50		
No	12	15.79			4	5.26		
*Lineages*			3.357	0.096			0.395	1
L2	20	22.73			6	6.82		
L4	2	7.14			1	3.57		
*Census register*			0	1				
Resident	17	16.83			3	2.97	2.952	0.209
Migrant	1	16.67			1	16.67		
*Health system delay*			0.818	0.516				
<14 days	16	18.39			3	3.44	0.109	0.569
≥14 days	2	10.00			1	5.00		
*Visiting hospital delay*			0.54	0.587			0.564	0.596
<14 days	7	20.00			2	5.71		
≥14 days	11	15.27			2	2.78		
*Source of patients*			4.44	0.218			14.966	**0.002**
Clinic visit due to symptoms	1	25.00			1	25.00		
Tracing	14	15.22			2	2.17		
Referral	1	50.00			1	50.00		
Health inspection	4	36.36			1	9.09		

Note: nine cases were missing information for both census register and health system delay; seven cases were missing information for the patient source. Pre-XDR-TB: preextensively drug-resistant tuberculosis; RR/MDR-TB: rifampicin-resistant or multidrug-resistant tuberculosis.

## Data Availability

The raw sequence data reported in this paper have been deposited in the Genome Sequence Archive (Genomics, Proteomics & Bioinformatics 2021) in the National Genomics Data Center (Nucleic Acids Res 2022), China National Center for Bioinformation/Beijing Institute of Genomics, Chinese Academy of Sciences (GSA: CRA008002), and are publicly accessible at https://ngdc.cncb.ac.cn/gsa.
